# *Candida albicans* exhibit two classes of cell surface binding sites for serum albumin defined by their affinity, abundance and prospective role in interkingdom signalling

**DOI:** 10.1371/journal.pone.0254593

**Published:** 2021-07-19

**Authors:** Claire Teevan-Hanman, Paul O’Shea

**Affiliations:** Faculty of Health & Medicine, Lancaster University, Lancaster, England, United Kingdom; Louisiana State University, UNITED STATES

## Abstract

Serum albumin binding to the yeast form of *Candida albicans* is described. Two populations of binding site are identified using two complementary spectroscopic techniques: an extrinsic fluorescent probe, 3-hexa-decanoyl-7-hydrocoumarin ([HEXCO) added to the C. albicans yeast cell surface that records the electrostatic surface potential and so responds to the surface binding of serum albumin and secondly a light scattering technique that reveals how albumin modulates aggregation of the yeast population. The albumin binding sites are found to possess different binding affinities and relative abundance leading to different total binding capacities. These are characterized as a receptor population with high affinity binding (Kd ~ 17 μM) but relatively low abundance and a separate population with high abundance but much lower affinity (Kd ~ 364 μM). The low-affinity binding sites are shown to be associated with the yeast cell aggregation. These values are found be dependent on the *C*. *albicans* strain and the nature of the culture media; some examples of these effects are explored. The possible physiological consequences of the presence of these sites are speculated in terms of evading the host’s immune response, biofilm formation and possible interkingdom signaling processes.

## Introduction

The complexity of the mutual interactions of invasive micro-organisms with their host is becoming much more evident with molecular-level characterizations of many inter-kingdom signaling processes. Their incidence appears ubiquitous with examples respectively between mammalian or plant hosts and bacteria [1], fungi etc and also between different pathogens within a single host [eg^2^, ^3^]. Similarly, the chemical nature of the biomolecular interactions that underlie inter-kingdom signaling events are equally extensive with examples ranging from gasses and ions to both polar and non-polar small molecules to larger molecules such as peptides and proteins [4].

In our studies of inter-kingdom signaling between invasive micro-organisms and mammalian hosts, we have characterised some of the manifold interactions mediated by a cross-talk between the so-called quorum sensing molecules and serum proteins and the host cell surfaces [5] as well as helping define the nature of bacterial LPS-host cell interactions [6]. Similarly, we have a longstanding interest in the physical properties of *Candida albicans* that relate to cellular interactions and particularly to cell-cell aggregation [7–9]. As part of these studies and within our broader research program directed at biomolecular interactions we have developed a comprehensive panel of spectroscopic and imaging-based technologies that illuminate molecular interactions with many kinds of biological surfaces using for example, plasmonics [eg 10], optical scattering techniques [eg 7] and fluorescence [eg 11, 12]. These tools can also aid identification of the spatial location of the molecular interactions on the surface of living cells and with model membrane surfaces [eg 13].

The rationale for the present study relates to questions of how *Candida albicans* may interact with proteins found in the serum of a prospective mammalian host in order to better define our understanding of the systems behavior of the host’s response to the pathogen invasion. This is ultimately also related to the mechanism of the cellular infection of the yeast. Several authors dating back over many years [14–16] acknowledged the vital importance of this step in the initiation of infection, however precise mechanisms are still not fully established. Specific receptors to various mammalian cell surface ligands have been documented such as receptors to laminin and vitronectin with several others depending on the cell type that becomes targeted [17]. Fungi also colonise artificial surfaces [18] and non-specific mechanisms based on simpler physical interactions also appear to be involved [8, 9]. It is clear, therefore, that adhesion processes are complex and diverse, allowing *C*. *albicans* to respond to the challenges presented by a variety of host environments.

Invasive Candidiasis remains a serious threat to human health [eg 19] and a better understanding of the respective responses of the host and the fungi may aid treatment strategies. *C*. *albicans* exploits the vascular network to establish infection throughout its host. The yeast form of the fungi therefore, encounters the constituents of the circulatory fluids such as serum proteins; the most abundant component of which is albumin [20]. The relationship of *C*. *albicans* with these proteins may well be complex [21]. Several proteins are thought to assist *C*. *albicans* in the invasion and colonization of the host [eg 22] and are implicated in the infection processes and host-immunological evasion [23, 24]. Introduction of albumin into cultures of *C*. *albicans* for example, has been shown to elicit the production of extracellular aspartyl proteinase. Similarly, Schurmann et al [25] demonstrated that a coating of serum albumin on polystyrene microspheres, afforded the particles resistance to phagocytosis by alveolar macrophages. In our laboratory we demonstrated that T and B lymphocytes exhibit differing capacities for bind albumin [26] and long ago it was suggested that albumin protect macrophages from proteinases released by *C*. *albicans* [27]. It would not be surprising therefore, if invasive micro-organisms possessed an ability to exploit a host’s plasma proteins although Page & Odds [28] suggested that there was little interaction of serum proteins with the yeast form of *C*. *albicans*. In addition, it is well known that serum protein interactions with therapeutic agents must be factored into the pharmacokinetic/dynamics when devising dosage [29]. Several Interesting questions therefore, relate to manifold roles serum proteins may play in the infection, adhesion and immunological evasion of fungal pathogens.

To define and address some aspects of the how serum proteins may interact with invasive yeasts we have employed two spectroscopic techniques. The first ultilizes an extrinsic fluorescent probe, 3-hexadecanoyl-7-hydrocoumarin (HEXCO). We previously characterised this probe to study the electrostatic properties of the surface of bacteria [30]. This fluorophore is included in our stable of such probes [see eg. 12, 31, 32] and aids the simple and reliable detection of changes of the cell surface electrostatic potential as a result of the binding of macromolecules [33]. The second optical technique in the present study makes use of 90° Rayleigh-Debye light scattering; details of the theory underlying this technique and some generic applications can be found in Davis *et al*. [34]. This light scattering technique enables the aggregation state of a fungal population to be assessed [7]. This yields a quantity termed the scattering gradient (ε_s_), a parameter that is analogous to the Beer-lambert wavelength-dependent extinction coefficient of an absorbing species.

The results of these studies indicate that *C*. *albicans* possess’ two classes of binding site on their cell surface for serum albumin. Based on their respective binding parameters these sites are suggested to be related to differing mechanisms of evading a prospective host’s defences and could well offer a new class of therapeutic targets.

## Methods

### *C*. *albicans*, strains and growth conditions

Four strains of *C*. *albicans* were used in the present study: MRL 3153, a standard laboratory reference strain, supplied from The British Mycological Reference Laboratory (Collingdale, London, U.K.) in freeze-dried form. The culture was revived with liquid medium (0.5ml) and used to innoculate agar slopes. [see 7, 8]. Strains GDH 2346 and GRI 681 were provided by GlaxoSmithKline (formerly SMKBeecham) U.K. on agar slopes. Strain NCPF 3153 from the National Centre for Pathogenic Fungi originally deposited by Professor Julia Douglas, University of Glasgow UK. The cultures were revived with Yeast Nitrogen Base (YNB) supplemented with 50mM glucose, 0.5ml. This was used to inoculate 200ml of the same medium and incubated for 48 hours at 37°C. Populations of cells were used to innoculate sterile agar slopes or petri dishes containing agar and incubated for 48 hours at 37°C. The dishes were then refrigerated at 4°C. Aliquots were harvested for subculture from the dished when required. These cells were incubated in selected growth media; RPMI 1640 containing 2% (w/w) glucose, YNB + 500mM galactose or YNB + 50mM glucose, for 18 hours at 37°C. Following harvesting, the fungi were pelleted using a microcentrifuge at high speed for 2 minutes. The supernatant was aspirated and discarded, then the cells resuspended in 1mM KCl + 1mM K^+^Hepes medium, pH 7.5 (or as appropriate). This process was repeated twice more leaving the cells suspended in fresh medium. Cell density was determined using a standard haemocytometer chamber and a light microscope, then adjusted to a desired concentration. This inspection also served as confirmation of the yeast rather than any hyphal form of the fungi.

### Preparation of serum albumin

Pure Human Serum Albumin (HSA), was obtained from Sigma in crystalline powder form. Solutions of required molarities were prepared using filtered 1mM KCl + 1mM K^+^Hepes medium, then adjusted for pH 7.5 (or as appropriate). Precise molarities were determined using a standard spectroscopic techniques for this protein determination [35] assuming a MWt of 66KD.

### 90° Rayleigh-Debye light scattering

Spectrofluorimetric determinations were performed with standard benchtop fluorimeters available in our laboratories: Horobin, Perkin Elmer or Shimatzu; specific the study regimes however, were performed only on the same fluorimeter and not collected with different instrumentation. The data were recorded, analysed (see equations below) and presented using appropriate software such as Biosoft, Prism, Origin etc. To assess the effect of HSA on the fungal aggregation state, Rayleigh-Debye light scattering was measured and recorded at 600nm and 90° to the incident irradiation also at 600nm.Yeast cells were prepared as described and suspended in filtered 1mM K^+^Hepes medium containing 1mM KCl, pH 7.5 at a concentration of 1x10^6^ cells/4.5μl. Serial additions of cells were made to the measuring cuvette (3 ml) containing either the filtered 1mM KCl + 1mM K^+^Hepes medium, pH 7.5 or HSA suspensions, pH 7.5. Any changes in scattering intensity was monitored against time until judged to be stable (see Fig 3). These experiments were performed for the three fungal strains cultured in each of the three growth media, and titrated with defined concentrations of HSA. This allows us to measure and compare yeast aggregation states using the scattering coefficient ε_s_, determined as the linear gradient.

### Interactions of HSA with *C*. *albicans* determined utilizing HEXCO fluorescence

Typically 3 x 10^7^ Cells were incubated for 1 hour at 37°C with 100μM HEXCO with gentle agitation in 1mM K^+^Hepes medium containing 1mM KCl, pH 7.5. The HEXCO stock solution of was stored in 95% ethanol at 2mg ml^-1^. using a MWt of 400.56. Unbound HEXCO was removed by microcentrifugation followed by careful aspiration of the supernatant. Labelled cells were then resuspended at appropriate concentrations in 1mM K^+^Hepes medium containing 1mM KCl, pH 7.5. prior to experimental use.

Fluorescence emission spectra of HEXCO-labelled *C*. *albicans*, were obtained after excitation at 395nm. The pH of 3x10^6^ cells/ml was adjusted with HCl or KOH as appropriate and the change in fluorescence emission spectra recorded. pK_a_ values for HEXCO were also determined in the presence and absence of 3x10^6^ labelled cells ml^-1^. The fluorescence intensity of the emission maximum (λ_max_) wavelength of 454nm following excitation at 395nm was identified as an appropriate measuring wavelength.

HEXCO-labelled *C*. *albicans*, were suspended in 1mM K^+^Hepes medium and aliquots of HSA were titrated into 7.5x10^6^ cells/ml with gentle mixing. The change in emission intensity was recorded at 454nm after excitation at 395nm. To obtain a broad range of HSA concentration, increases in aliquot concentrations were made. Initially additions of 0.5μM HSA were added rising to 81.1μM. although the whole physiological range of HSA concentration was studied.

### Equations used for the data analysis

Data were fitted to the following equations. A best-fit was determined based on R^2^ following lea-squares based iterations to each of the following equations:

$$y=B_{ma}{}_{x}.X/K_{d}+X$$ (1)

$$\begin{array}{l} y=[B_{max[site\;1]}.X/K_{d–site\;1}+X]\\ \;+[B_{max[site\;2]}.X/K_{d–site\;2}+X] \end{array}$$ (2)

$$y=[B_{max}{}^{h}.X^{h}/K_{d}{}^{h}+X^{h}]$$ (3)

Where *y* is the observed spectroscopic signal, *x* is the cumulative concentration of added species (yeast or HSA), K_d_ is the apparent affinity constant [in concentration units], B_max_ is the 100% capacity of the surface (in arbitrary units of fluorescence or light scattering AU:). The exponent h represents an index of cooperativity.

## Results

### The effect of serum albumin on the aggregation state of a *C*. *albicans* population

**Fig 1:**
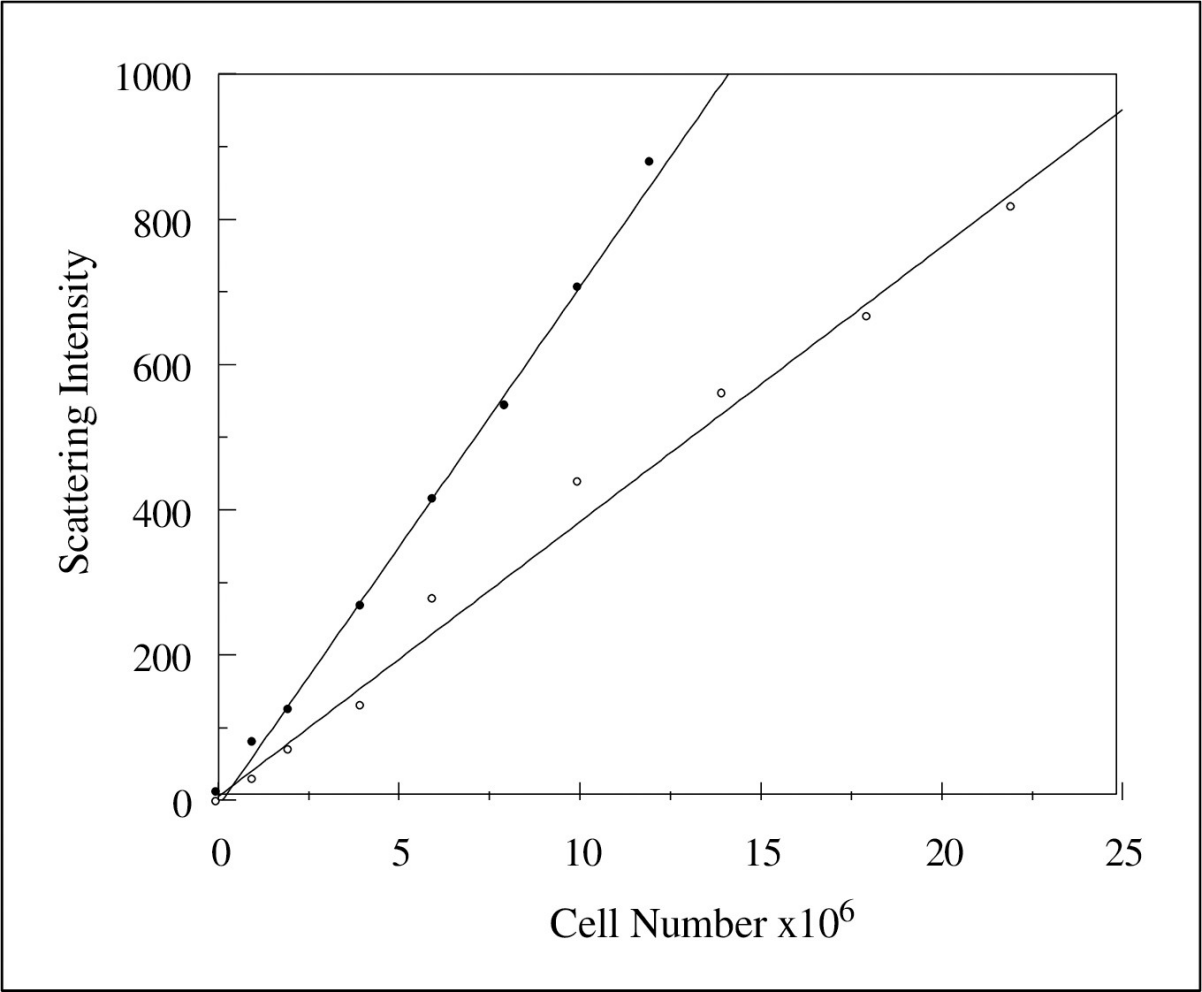
The effect of 172μM HSA on the 90° Rayleigh-Debye light scattering of *C*. *albicans* [strain MRL 3153 cultured in RPMI 1640] at 600nm following excitation at 600nm. Experiments were performed in either 1mM KCl supplemented with 1mM K^+^ Hepes, pH 7.5 ● or 1mM KCl supplemented with 1mM K^+^ Hepes and 172μM HSA ◯. The respective scattering gradients [ε_s_] are 71.5 ± 3.9 and 37.8 ± 3.4.

90° scattered light intensity was recorded in real-time following serial additions of *C*. *albicans* as previously described [7, 34] an example of which is plotted in Fig 1. The plot illustrates the linear nature of the light scattering changes and allows calculation of a scattering coefficient, ε_s_, [34]. In earlier studies, the scattering technique was used to determine if growth conditions may affect the ability of the fungi to aggregate [7]. In this previous work, the hydrophobic nature of the fungal surface was also correlated with the ability of the yeast population to aggregate.

The introduction of human serum albumin (HSA) to a suspension of yeast cells leads to a reduction in the degree of light scattering but the gradient with respect to the yeast concentration remains is linear and so a scattering gradient can still be determined. The reduction in ε_s_. evident due to the addition of HSA shown in Fig 1. suggests that it promotes self-aggregation of *C*. *albicans*. The respective scattering gradients (ε_s_) are in the absence of HSA 71.5 ± 3.9 and following the addition, 37.8 ± 3.4, equivalent to an increase in aggregation by 47.1% due to the interaction of HSA at the concentration used in the example shown in Fig 1.

In earlier work [7] we found that *C*. *albicans* possessed an inherent ability to aggregate and this varied with both the strain and nature of the growth media. The rationale for the choice of the growth media in our current study and shown in Table 1 were based on our original studies in which we surveyed a broad range of growth conditions (and Candida variety) to explore their effects on the fungal surface hydrophobicity [7] and surface charge [8]. Significant difference were found in the surface hydrophobicity as related to aggregation whereas smaller differences appear to be evident in terms of the albumin binding parameters even in the case of what would be considered excessive concentrations of a nutrient such as with the Galactose supplement in Table 1. It is worth noting as well that the studies with the RPMI media were specifically with the yeast form of the Candida although we cannot discount that some germ tube formation may be taking place as this media is known to elicit such formation [35, 36]. A follow-up study to the present study involves imaging which has facilitated identification of the locality on surface of the binding reactions and there are indications that the albumin binding is not homogeneous. Further investigations in our current study as detailed in Table 1 indicate that the aggregation promoting effect of HSA is also similarly ubiquitous with the extent modulated by the growth media and strain. Overall, for the three strains grown under the different culture media, the average decrease in ε_s_ and therefore HSA-dependent increase in aggregation at 30μM HSA, is 21.4% and at 100μM HSA, it is 37.4%. HSA still appears to promote aggregation therefore but this ability does not seem susceptible to major changes as the result of different growth media or the strain genetics. In the next sections a more detailed characterization of the nature of this behavior is explored.

**Table 1:** A summary of the effect of HSA on the 90° Rayleigh-Debye light scattering of 3 strains of *C*. *albicans*.cultured in 3 different growth media.

Fungal Strain	Growth Medium	Buffer Control [+/- SEM.]	% gradient difference for 30μM HSA [+/- SEM.]	% gradient difference for 100μM HSA [+/- SEM.]
**MRL 3153**	**RPMI 1640**	57.1 ± 1.1	-23.6 ± 2.9	-39.4 ± 3.3
**MRL 3153**	**YNB + 50mM Glucose**	55.9 ± 0.6	-20.2 ± 1.2	-34.5 ± 1.8
**MRL 3153**	**YNB + 500mM Galactose**	49.1 ± 0.8	-17.9 ± 1.0	-37.3 ± 4.4
**GDH 2346**	**RPMI 1640**	49.0 ± 0.9	-20.4 ± 1.2	-36.3 ± 2.6
**GDH 2346**	**YNB + 50mM Glucose**	58.3 ± 1.1	-24.7 ± 1.1	-37.6 ± 3.0
**GDH 2346**	**YNB + 500mM Galactose**	54.4 ± 0.7	-21.5 ± 1.3	-40.4 ± 3.2
**GRI 681**	**RPMI 1640**	96.2 ± 4.4	-22.5 ± 1.3	-39.0 ± 2.7
**GRI 681**	**YNB + 50mM Glucose**	79.4 ± 1.5	-21.9 ± 0.7	-34.8 ± 3.4
**GRI 681**	**YNB + 500mM Galactose**	74.8 ± 0.9	-19.8 ± 0.9	-36.9 ± 3.3

Quantities are quoted for the measurements in 1mM KCl supplemented with 1mM K^+^ Hepes control and % scattering gradient differences, relative to the HSA-free control, shown for 30μM HSA and 100μM HSA.

### The cooperative nature of the HSA-dependent change of the aggregation state of *C*. *albicans*

**Fig 2:**
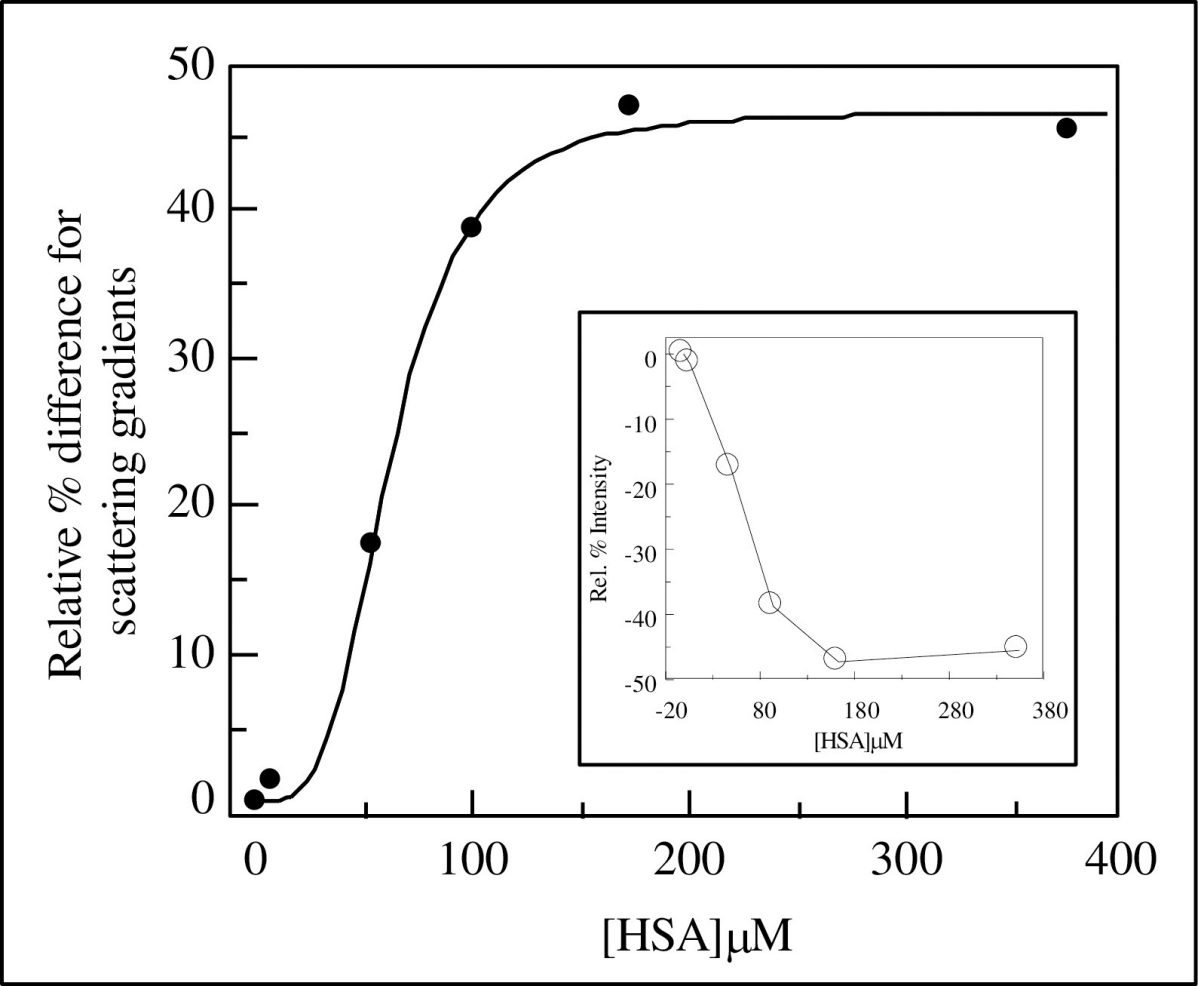
Plot of the scattering gradients (ε_s_) versus HSA concentration. 90° Rayleigh scattering was performed by titrating *C*. *albicans* (MRL 3153 grown in RPMI 1640) into filtered 1mM KCl +1mM K^+^ Hepes at pH 7.5 supplemented with 0μM, 7.52μM, 53.4μM, 172μM, or 374μM HSA. The increase in emissions intensity at 600nm was recorded after excitation at 600nm. For each HSA concentration, the scattering gradients were determined. The gradients were manipulated by calculating the percentage difference relative to the 0μM HSA gradient, then multiplying by -1. These values were then plotted against HSA concentration and a Hill equation fitted. n = 3.5 ± 0.9 and the Vmax. 46.6 ± 4.2 The inset shows the original percentage data.

The concentration profile of the changes of Scattering coefficients for *C*. *albicans* were explored over the physiological range of HSA concentration. The resultant data were fitted to a number of models but linear or hyperbolic relationships proved to yield very poor fits whereas a sigmoidal relationship appeared the most appropriate mathematical description as shown in Fig 2. The equations describing these models are listed above [as Eqs 1–3] with Eqs 1 and 2 as a simple hyperbolic relationships and Eq 3 defining a co-operative model. Eq 3 includes an exponent which we define as a co-operativity coefficient parameter [h] equivalent to the Hill coefficient associated with co-operative interactions. A value of 3.5 ± 0.9 for *h*, with a capacity (B_max_)of 46.6 ± 4.2 yielded the best fit illustrated in Fig 2. This indicates that the nature of HSA-dependent aggregation binding to *C*. *albicans* is positively cooperative. It also indicates that the maximum effect of HSA is an increase in the aggregation ability by 46.6% ± 4.2.

Interpretation of the *h* parameter as an indicator of the molecular nature of the co-operativity mechanism associated however, can be ambiguous. Thus a number of explanations that reside in the molecular nature of the HSA-dependent aggregation phenomenon will require more advanced experimentation and will form the focus of further studies. For the moment, a simple interpretation suggests that up to 3 sets of HSA binding sites on the fungal surface are associated with the yeast-yeast aggregation behavior.

## HSA binding to *C*. *albicans* as revealed using HEXCO as a fluorescence probe

### The mode of action of HEXCO as an indicator of molecular binding to the fungal cell surface

The measurement rationale we have developed to monitor molecular binding to biological surfaces is as follows: Utilising fluorophores whose fluorescence is strongly affected by the protonation state of the fluorescence moiety by locating the on a biological surface the fluorescence yield can be affected by the net charge density on the surface. Thus changes of the charge density following the binding of a charged molecule (such as albumin) offers a method of detecting the binding events. This phenomenon is a consequence of the Boltzmann effect and involves a change in the pK of the fluorescence moiety due to the change in the surface charge density. In a purely isotropic aqueous environment the HEXCO fluorophore exhibits a fixed pK. The protonation state of the fluorescent moiety modifies the fluorescence yield and so any changes of pK at a constant ambient pH will be observed as changes of fluorescence due to consequent changes of the protonation state. When located on the fungal cell surface because of the proximity of charges on the surface, the HEXCO pKa becomes shifted. This phenomenon is well characterized and can be implemented with a number of different probe systems designed to target various biological surfaces but all possessing the same kind of response. Examples explained and outlined in a number of biological systems [reviewed in 37] ranging from the mammalian cell surface [12] to microbial surfaces [11, 30, 31].

Fluorescence signal changes in these systems may have two different origins: i] by changes of the surface charge density (either positively or negatively)–this can be achieved by charged molecules becoming bound to the fungal surface leading to pK shifts of HEXCO OR ii] by protonation or deprotonation of the HEXCO on the cell surface. Thus measurement by pH titration to determine the HEXCO pK of the labelled candida being shifted as compared to the pK of HEXCO in an entirely aqueous environment is confirmation of a successful labelling method. The pH titration is undertaken in which fungi are suspended in media of very low pH buffering capacity in order to minimize large acid or base additions.

Following identification of the conditions for successful labelling of the fungal surface with some optimization of the protocol, it is not necessary to undertake a pH titration on every preparation of HEXCO-labelled Candida because fluorescence changes following the addition of charged molecules to the fungal suspension cell surface at constant ambient pH can only lead to such changes through a pK shift due to the binding of the added molecules. Such changes do not take place to the fluorophore in an aqueous medium in the absence of the fungi. The fluorescence of a sample of each preparation of HEXCO-labelled Candida may be challenged with a molecule that we know to bind to the negative cell surface of Candida. Typically a few mM of Calcium ions (as CaCl_2_) is a useful quality control measure of the ability of the labelled preparation to detect the binding of charged molecules to the fungi through such fluorescence changes.

The data showing stepwise fluorescence changes due to the addition of a charged molecule as described below for albumin therefore results from an increase of negative charge to the cell surface that is interpreted to indicate albumin becoming bound to the surface. A plot of the change versus the concentration therefore yields a binding constant in concentration units. It is also possible to determine the total number of albumin molecules that have become bound but that is rather more involved and beyond the scope of the current paper as the location of the binding on the cell surface is also heterogeneous but this spatial imaging information will form the basis of the next paper in this series. The characterisation process described above therefore is shown in the following section.

## Characterisation of the fluorescence properties of HEXCO-labelled-*C*. *albicans*

**Fig 3:**
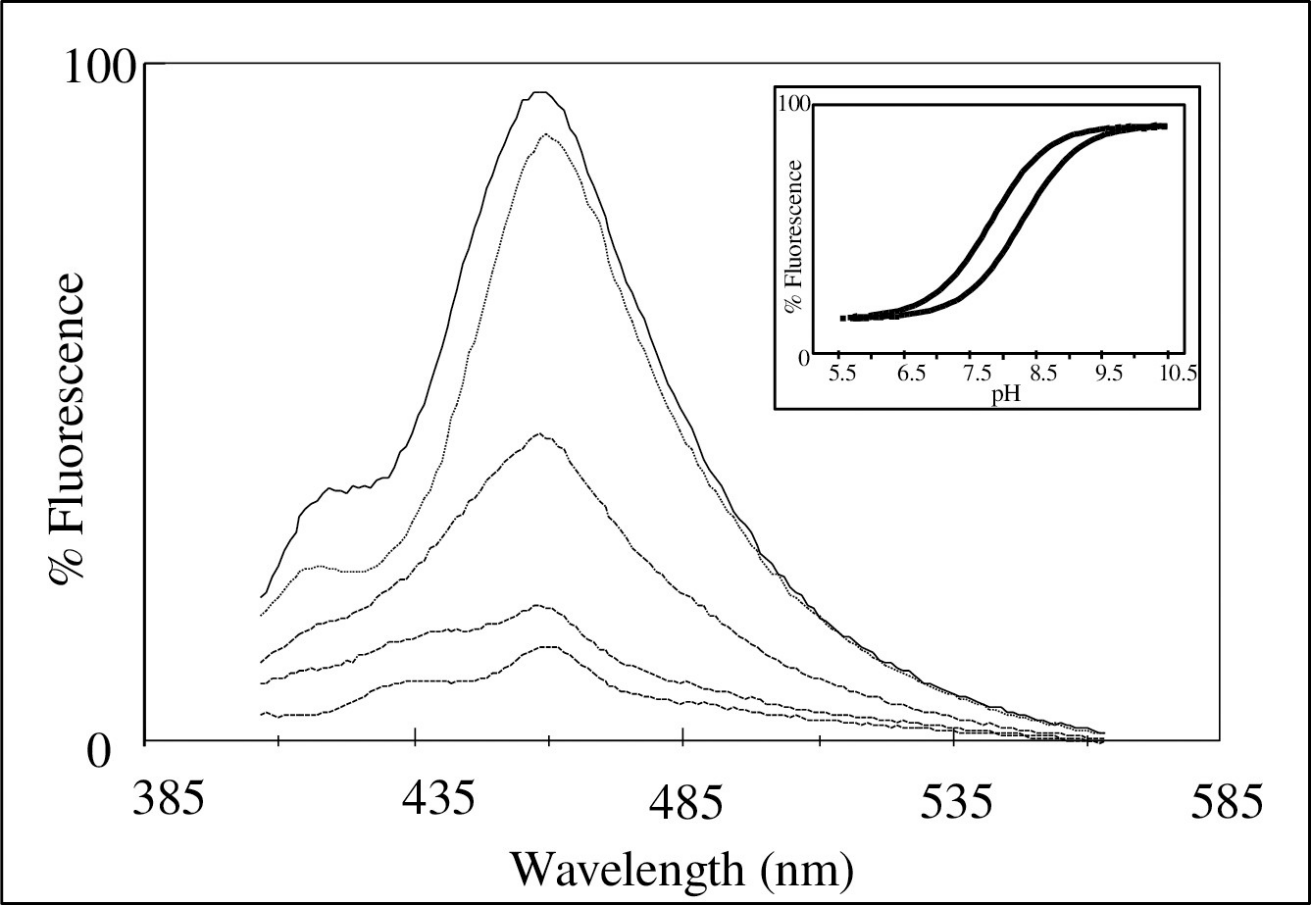
The fluorescence emission spectrum of HEXCO labelled *C*. *albicans* (NCPF 3153 grown in RPMI 1640), at increasing pH [5.46, 5.95, 7.43, 8.63, 9.34 respectively]. The cells were incubated for 1 hour at 37°C with 100μM HEXCO with gentle agitation. After removal of unbound HEXCO, spectra of these cells in 1mM KCl were obtained after excitation at 395nm, λ_max._ was found to be 454nm. The inset shows the effect of pH on the fluorescence emission λ_max._ of free HEXCO and HEXCO labelled cells. pK_a_ values for HEXCO were determined and found to be 7.6 ± 0.1 in the presence of *C*. *albicans* and 8.3 ± 0.1 in their absence.

**Fig 4:**
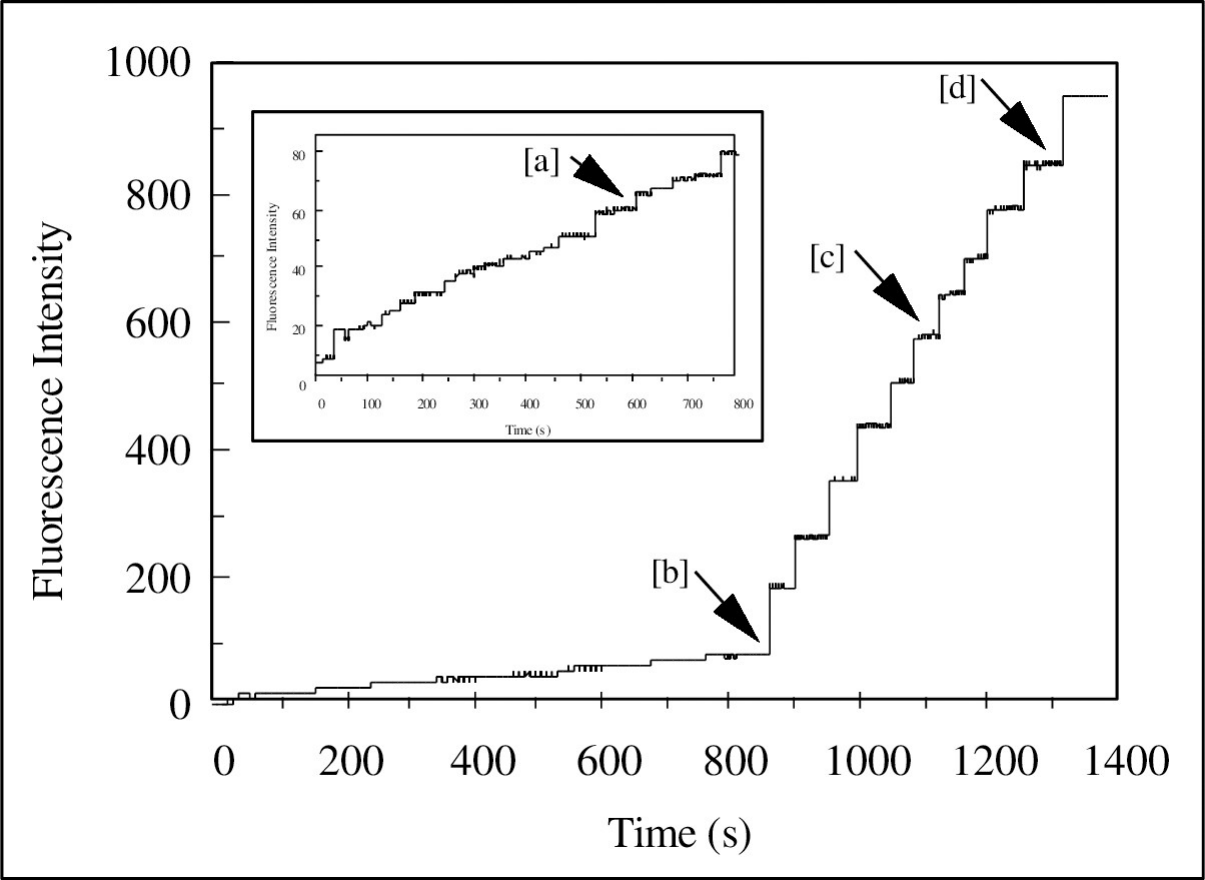
Example of the measurement of the time dependence of the fluorescence intensity following additions of [HSA] on HEXCO labelled *C*. *albicans*. HSA was added at the times indicated by the arrows. Initially serial additions of aliquots of 0.5μM HSA were added followed by 0.95μM at [a], 28.3μM at [b], 43.6μM at [c] and 81.1μM at [d].

Fig 3 shows the fluorescence emission spectrum of HEXCO-labelled *C*. *albicans* in 1mM KCl, 1mM K^+^ Hepes, after excitation at 395nm. The intensity of fluorescence increases with increasing pH and a clear peak (λ_max._) for each pH can be found at 454nm. Following this, pK_a_ values were determined for HEXCO and the pK shift confirms the binding of HEXCO to *C*. *albicans*. From the characterisation of HEXCO in Fig 3. further studies of the interaction of HSA with *C*. *albicans* (MRL 3153 cultured in RPMI 1640) could be undertaken. HEXCO *-labelled C*. *albicans* were titrated with HSA with examples of the time-dependence of the fluorescence emission changes illustrated in Fig 4. The fluorescence was found to change very rapidly with each addition of HSA and does not occur in the absence of the yeast cells.

**Fig 5:**
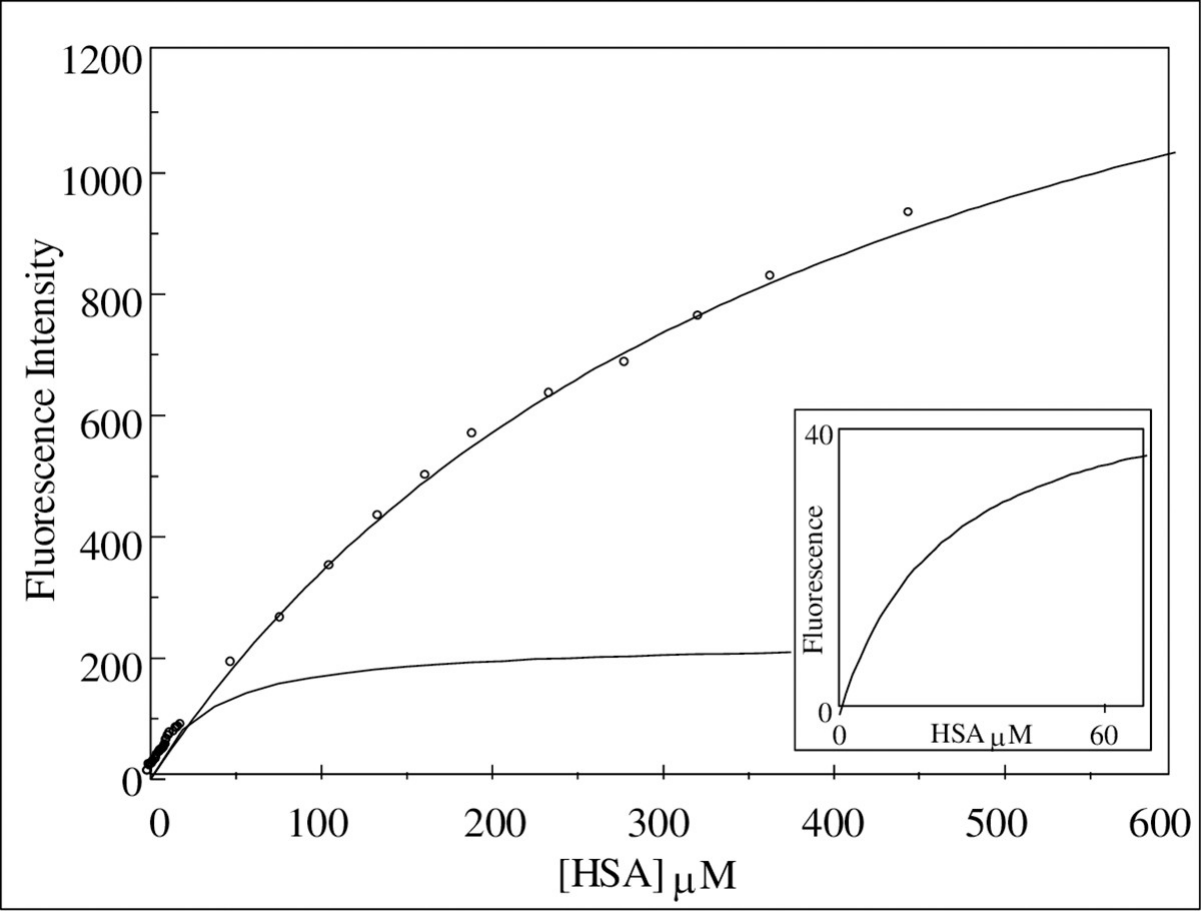
The effect of increasing HSA concentration on HEXCO labelled *C. albicans* (MRL 3153 grown in RPMI 1640). HSA was titrated into 1.5x10^6^ labelled cells ml^-1^ suspended in 1mM KCl supplemented with 1mM K+ Hepes at pH 7.6. The fluorescence emission was recorded at 454nm following excitation at 395nm as indicated by the data points. These were fitted as indicated by the solid lines to binding isotherms. The main plot exhibits two overlaid binding curves: a high affinity site one with Kd = 17.1 ± 1.9 μM and Capacity = 98.5 ± 7.6 and the other, a low affinity site with Kd = 364.1 ± 36.2 μM and Capacity = 1547.0 ± 93.9. The inset plot shows the with the fluorescence measurements made over the range 0–60 μM HSA. indicating the higher affinity binding profile defined by the binding affinity of Kd = 17.1 ± 1.9 μM.

The net signal changes of the fluorescence can be plotted against the HSA concentration as shown in Fig 5 and fitted to a number of different binding models (Eqs 1–3). These are shown as solid lines running through the data points in Fig 5. The best-fit to these models indicated that although the binding relationships were relatively simple (exhibiting hyperbolic binding) it was necessary to combine two such binding processes each with a different set of binding parameters (affinity and capacity) as described by Eq 2. Thus the Candida cell surface appears to possess a combination of two independent binding sites each exhibiting a simple hyperbolic binding process. The binding profile could be defined therefore, by a combination of two simple hyperbolic processes to best-describe the observed binding isotherms. The binding profile was characterized by a high-affinity, low abundance site exhibiting a Kd of 17.1 ± 1.9 μM and a Capacity of 98.5 ± 7.6 AU and the second, a lower affinity but high abundance site with a Kd of 364.1 ± 36.2 μM and a Capacity of 1547.0 ± 93.9 AU.

Table 2 indicates that the presence of high and low affinity HSA binding sites appear to be present in the each *C*. *albicans* strain. There are differences between the strains and an influence that is dependent on culture media, particularly with the higher affinity binding site. When the fungi are cultured in RPMI 1640, GRI 681 has the greatest Capacity and Kd value, for high affinity binding, followed by NCPF 3153. GDH 2346 has the smallest Capacity whereas MRL 3153 has the smallest Kd. In galactose medium, the Capacity is reduced and the Kd increases slightly. This suggests the presence of specific receptors whose expression may vary with strain and growth media.

**Table 2:** A summary of the trends obtained from HSA binding equations. The results shown are % differences relative to those values for strain MRL 3153 grown in RPMI 1640.

Strain and growth medium	High Affinity [0−20 μM HSA]		Low Affinity [0−446 μM HSA]	
	Capacity	Kd μM	Capacity	Kd μM
MRL 3153 in RPMI *	98.5 ± 7.6	17.1 ± 1.9	1547.0 ± 93.9	364.1 ± 36.2
GDH 2346 in RPMI	- 10.2% ± 1.02	+ 35.2% ± 6.3	- 22.1% ± 2.1	- 40.5% ± 7.7
GRI 681 in RPMI	+ 153.4% ± 33.2	+325.9% ± 87.9	+ 8.0% ± 1.0	+ 15.7% ± 3.2
NCPF 3153 in RPMI	+ 43.3% ± 5.23	+ 125.9% ± 23.7	+ 24.1% ± 2.1	+ 41.4% ± 5.7
MRL 3153 in Galactose†	- 39.1% ± 5.3	+ 19.0% ± 3.3	-	-

There are differences between the low affinity binding sites associated with the different strains, although they are not as significant as with high affinity binding. NCPF 3153 has the largest Capacity and Kd, followed by GRI 681, then MRL 3153. GDH 2346 has both the lowest Capacity and Kd value.

## Discussion

The mechanisms by which *C*. *albicans* may elude a prospective Host’s immune system is clearly of both fundamental and medical importance and the current work offers some insight into potential roles played by serum albumin in mammalian systems (Human). We began to address this broader question many years ago in studies that attempted to correlate yeast cell surface hydrophobicity with the ability of the *C*. *albicans* to aggregate using the Rayleigh-Debye light scattering technique combined with fluorescent sensors of surface polarity [7]. This light-scattering technique offers a simple but sensitive methodology in which the aggregation state of fungi can be measured and particularly by generating a metric through which comparisons between fungal strains, culture conditions and environmental conditions can be easily made. In terms of the latter, clearly one consideration would be the presence of plasma proteins and how the fungi may perceive them.

This latter question was also addressed many years ago by Page & Odds [28] who concluded that although some serum proteins such as albumin and fibrinogen could become bound to C. albicans, this only appears to occur with the germ tubes and not to the yeast cell surface. This was clearly at odds with some of our current observations and merited further inspection of any difference between the experimental protocols of Page & Odds [28] and our present work which focusses solely on the yeast form of *Candida*. These authors as part of an immunofluorescence study of the plasma protein-binding made careful measurements and went to some length to remove any unbound protein from the fungi. In particular, they state that “*cells were pelleted in a microcentrifuge and resuspended in PBS three times to remove unbound proteins then finally resuspended in PBS”* for immunofluorescence measurements of plasma protein binding [from Page & Odds 28]. Under these circumstances however, we would expect that reversibly-bound albumin would be essentially ‘*washed off’* the fungal surface. Thus, the previous work of Page & Odds [28] is not contradictory to our present study (and we are not contradicting their study) as the albumin-binding processes we observe are not effectively directed at studies of HSA that is essentially irreversibly bound. In fact, in a follow-up study to the work we report in the present paper we will illustrate the spatial location of HSA about the yeast cell surface using immunofluorescence and FITC-labeled HSA using fluorescence imaging techniques. It is clear from these studies that are complementary to the work of Page & Odds [28], that HSA binds about the cell surface in particular regions (manuscript in preparation).

The information gathered from scattering data illustrated the ability of HSA to promote self-aggregation of *C*. *albicans*; (Fig 1. inset). The implications of this are that in yeast form *C*. *albicans* possesses a predisposition to self-aggregate that is augmented by the presence of HSA. This appears true for all the strains examined independent of their culture media as shown in Table 1 and even at low concentrations HSA may promote aggregation.

The relationship between the HSA binding properties and the fungal aggregation profile implies that in human plasma, the level of yeast aggregation due to HSA would be at a maximum. Concentrations of HSA in interstitial fluids and other physiological compartments however can exist at significantly lower levels than in plasma [37] and also vary quite markedly during both normal physiology and under many different pathophysiological conditions. The level of aggregation in these environments therefore may well be much less evident than in plasma.

The nature of HSA-dependent aggregation appears to be positively cooperative indicating that after initial HSA binding, further aggregation is promoted as illustrated in Fig 2. This may be due to initial specific, perhaps receptor-mediated binding, followed by more rapid and less discriminate binding and crosslinking leading to aggregation. Crosslinking could occur, for instance, by hydrophobic or electrostatic attraction between bound HSA molecules on individual yeasts, creating a yeast-HSA-HSA-yeast complex or attraction between a bound HSA-yeast and another yeast, giving a yeast-HSA-yeast complex. The involvement of hydrophobic attractions in Candida adherence, to self, host tissue and non-biological interfaces, has been explored over a number of years in many laboratories [7–9, 11, 18, 38–43] to more recent studies [44] and remains a viable target for therapeutic intervention. The nature of such intervention could well be based on specific interference with the physical interaction mechanisms. In earlier studies we demonstrated that a number of small molecule reagents can interfere selectively with particular molecular forces involved in bother specific and non-specific binding reactions [10]. In particular the behavior of HSA was explored and a number of conditions were identified that modulated its specific and non-specific binding reactions. It remains to be seen whether these conditions may also have some use in targeting HSA- *C*. *albicans* interactions as part of a novel medical therapy strategy. With this possibility in mind, we consider the further characterization of the HSA- *C*. *albicans* interaction in the final part of the present paper.

Interpretation of the HSA-binding data to the yeast cell surface indicates the presence of two classes of binding site with different affinities; a low-abundance, high-affinity site and a separate high-abundance, low-affinity site. For the standard laboratory strain, MRL 3153, the Kd of the high-affinity site is 17.1μM and the low affinity site is 364.1 μM (Table 2).

On the basis of the forgoing studies, the surface of *C*. *albicans* in a typical human plasma environment may be expected to be saturated with HSA (albeit not irreversibly). There are several possible advantages that such an albumin coating may confer particularly during the events that underlie host invasion. HSA may act as a ‘molecular camouflage’ against neutrophil and other immuno-attack. Alternatively, yeasts may recognize serum albumin or albumin receptors, already on a tissue surface and use this to target particular tissues. Similarly, it has been suggested that early steps in pathogenesis is associated with the morphogensis from yeast to a hyphal state and that the latter may be more susceptible to phagocytic attack [45]. The relationship acknowledged here between serum albumin and yeast cells begins to offer a mechanism by which *C*. *albicans* may remain in unicellular form due to enhanced protection until such time as conditions for tissue invasion become more favourable.

The foregoing paragraphs outline further indications of the growing understanding of serum protein-Candida interactions in which the fungal behavior is modified by interaction with their molecular environment with some acknowledgments this is also related to pathological mechanisms in the event of host invasion. Such interaction is part of the growing body of knowledge related to inter-kingdom signaling. Many studies have explored the molecular physiology of the consequences of such interactions on morphogenesis and pathogenicity. These studies have dissected the genetic and signaling responses to interaction of the fungi with various surfaces and serum proteins. In the present study the interaction and broad effects of albumin at a number of concentrations have been characterized but the consequent effects on the intracellular signaling processes are yet to be resolved. Nevertheless, it is apparent that the albumin receptor systems do lead to different responses. These may range from ‘simply’ responding to fatty-acids carried by the host’s albumin as for example reported in [46] that have profound effects on morphogenesis to direct signaling effects due to occupation of as yet unidentified character of the albumin ‘receptor’. The next phases of this work therefore will utilize the effects of different albumin concentrations to dissect any concentration dependence of the intra-cellular signaling systems. We have not undertaken this as yet as we are in the process of reporting the surface localization of the albumin binding receptors as well as their identity as they may well have other functions. Each of these parameters may well be involved in different systems or responses and the coarse behaviour reported in the present paper needs to be fully characterised prior to deeper molecular inter-kingdom signaling processes.

Following this foregoing discussion it also seems highly relevant to consider the pioneering work of Klotz and coworkers [47] in which they studied the ability of yeast cells to adhere to 90-μm-diameter polyethylene glycol beads coated with a 7-mer peptide from a library of 197 unique peptide-beads. These adhesive beads carried a three-amino-acid sequence motif (τφ+) that possessed a vast combinatorial potential. These authors outlined what they termed a **degenerate recognition system** conferring on the fungi the means of adhering to a multitude of proteins and peptides associated with prospective hosts for either commensal or pathogenic behaviour. Whilst the binding characteristics of a population of beads equipped with multiple potential ligands cannot be directly compared to our present study in terms of the thermodynamic parameters such as equilibrium constants for binding (as the beads would exhibit the thermodynamic ‘Chelate effect’ leading to an apparent greater binding affinity), the Klotz et al [47] study illustrates that host peptide/protein binding is a significant feature of the fungi’s repertoire for targeting a prospective host.

In view of the work described by Klotz and coworkers above, it is also worth mentioning that in the event our HSA preparations contained a significant amounts of denatured or fragmented albumin they may be recognized by receptor systems on the fungal cell surface and their binding would be reported by the HEXCO fluorescence measurement system. We have long experience of working with albumin however and routinely check our preparations for denaturation using SDS-PAGE and the spectroscopic tools we have developed to study the structural changes of albumin [48].

Another benefit to the yeast of an albumin ‘coating’ and an albumin-promoted yeast aggregation relates to antifungal therapy with drugs such as Amphotericin or Fluconazole. Thus, a network of HSA-yeast-HSA-yeast emboli which are at first targetted as part of a treatment regime for some form of fungemia associated with Candidiasis by such drugs ultimately must be terminated due to host-drug toxicity. This limitation to the exposure period of any therapy means that the fungal components within the interior of the emboli may still remain viable enabling rapid re-establishment of infection underlying persistent fungemia [49]. It is possible, therefore, that as well as promoting emboli of fungi, the HSA coating in itself could be considered a physical impediment to the drug molecules as there is a large body of literature associated with the pharmacokinetics/dynamics of drug molecules and the effects of albumin on these important therapeutic parameters [50, 51].

## Supporting information

S1 File (PDF)
