# Supplementary material for: Candida albicans exhibit two classes of cell surface binding sites for serum albumin defined by their affinity, abundance and prospective role in interkingdom signalling
Source: PLoS One. 2021 Jul 19;16(7):e0254593. doi: 10.1371/journal.pone.0254593 (PMC8289007; doi:10.1371/journal.pone.0254593)
Supplement: S1 File — (PDF) [file pone.0254593.s001.pdf]

Candida Scattering data +/- HSA

Fig. A. (For final picture, see Fig. B. below.)

The cooperative nature of the effect of Human Serum Albumin on the aggregation state of *C. albicans*

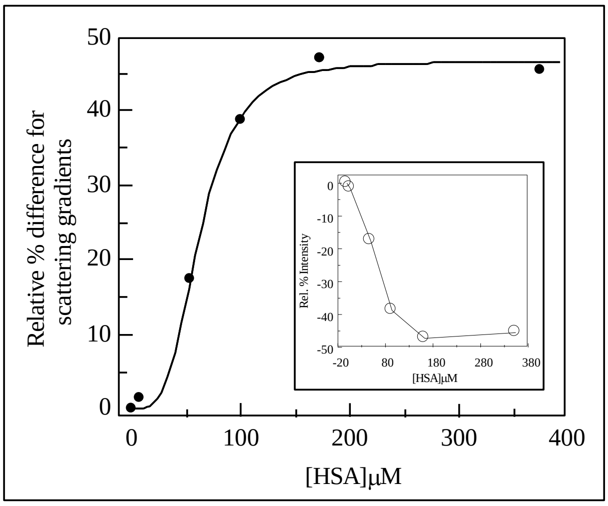

Plot of the scattering gradients (ε) verses HSA concentration. 90° Rayleigh scattering was performed by titrating *C.albicans* (MRL 3153 grown in RPMI 1640) into filtered 1mM KCl at pH 7.5 supplemented with 0μM, 7.52μM, 53.4μM, 172μM, or 374μM HSA. The increase in emissions intensity at 600nm was recorded after excitation at 600nm. For each HSA concentration, the scattering gradients were determined. The gradients were manipulated by calculating the percentage difference relative to the 0μM HSA gradient, then multiplying by -1. These values were then plotted against HSA concentration and a Hill equation fitted. **n = 3.5 ± 0.9 and the Vmax. 46.6 ± 4.2 the goodness of fit was 1.0 = a perfect fit.** The inset shows the original percentage data.
As n =3.5 this indicates that there is more than a 1:1 ratio for the binding of HSA to the fungal surface.

Table showing the manipulation of data for the plot above. For reference only not for quoting.

[HSA]μM	Percentage	x % by -1
0	0	0
7.52	-1.5	1.5
53.4	-17.3	17.3
100	-38.7 (ave.)	38.7
172	-47.1	47.1
374	-45.3	45.3

Interpretations and Analysis

- " A sigmoidal curve is diagnostic of a cooperative interactions between a structures small molecule binding sites; that is, the binding of one small molecule affects the binding of others." eg. in the case of hemoglobin, "the binding of O_2 increases the affinity of Hb for the binding additional O_2."

- If E = protein consisting of *n* subunits that can each bind a molecule S, (ligand), then;

$$E + nS \leftrightarrow ES_n. \qquad \text{equation [1]}$$

If the ligand binds with infinite cooperativity, i.e. no observable intermediates, ES_1, ES_2, etc. The dissociation constant for this reaction is

$$K = \frac{[E][S]^n}{[ES_n]} \qquad \text{equation [2]}$$

the Hill equation is described in terms of fractional saturation and eventually becomes:

$$Y_s = \frac{[S]^n}{K+[S]^n} \qquad \text{equation [3]}$$

this describes the degree of saturation of a multisubunit protein as a function of ligand concentration. However, *n* (the Hill constant) may be taken to be the degree of cooperativity among interacting ligand-binding sites rather than the number of subunits per protein. If **n = 1**, equation [3] is a hyperbola and the ligand binding is **noncooperative.** A reaction with **n > 1** is **positively cooperative**: ligand binding increases the affinity of E for further ligand binding. Conversly, if **n < 1**, the reaction is **negatively cooperative:** ligand binding reduces the affinity of E for further ligand binding.

- The Hill plot; $\log[Y_s/(1-Y_s)]$ versus log [S], has a slope of *n*.

- So for the fungi; n = 3.5 which is positively cooperative indicating that as a molecule of HSA binds to the fungal surface, it encourages further HSA to bind. Could the initial binding be specific receptor mediated binding causing some sort of change either conformationally or chemically (by expression of a signal), which then encourages subsequent non-specific binding ie. hydrophobic interaction? Also as each individual fungal particle is enveloped with HSA, other fungal particles see coated fungi as a source of HSA and so bind creating crosslinking.

Fig. B. Final Picture Used In the Paper. Inset is scattering data

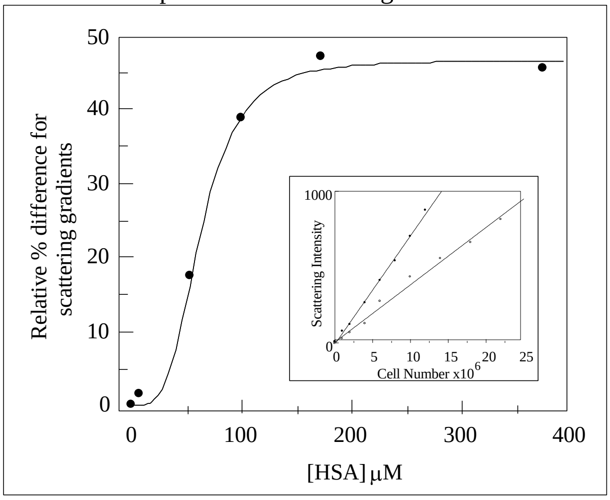

Plot of the scattering gradients (ε_s) verses HSA concentration. 90° Rayleigh scattering was performed by titrating *C.albicans* (MRL 3153 grown in RPMI 1640) into filtered 1mM KCl +1mM K^+ Hepes at pH 7.5 supplemented with 0μM, 7.52μM, 53.4μM, 172μM, or 374μM HSA. The increase in emissions intensity at 600nm was recorded after excitation at 600nm. For each HSA concentration, the scattering gradients were determined. The gradients were manipulated by calculating the percentage difference relative to the 0μM HSA gradient, then multiplying by -1. These values were then plotted against HSA concentration and a Hill equation fitted. **n = 3.5 ± 0.9 and the Vmax. 46.6 ± 4.2 the goodness of fit was 1.0 = a perfect fit.** The inset shows the original percentage data.

The insert, The effect of 172μM HSA on the 90° Rayleigh-Debye light scattering of *C. albicans* (strain MRL 3153 cultured in RPMI 1640) at 600nm following excitation at 600nm. Experiments were performed in either 1mM KCl supplemented with 1mM K^+ Hepes, pH 7.5 ● or 1mM KCl supplemented with 1mM K^+ Hepes and 172μM HSA ○. The respective scattering gradients (ε_s) are 71.5 ± 3.9 and 37.8 ± 3.4, which is equivalent to a reduction of (ε_s),or increase in aggregation, by 47.1% due to the presence of HSA.

The two original figs. for scat grad. versus [HSA], + Hill plot. and is % scat grad.

Original Picture - Inset Showing actual data.

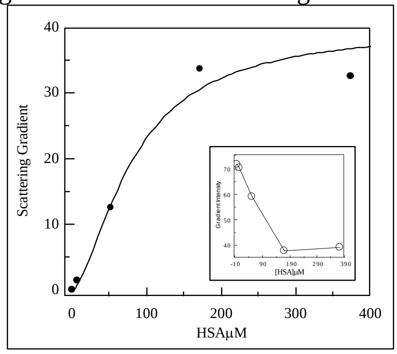

Plot of the scattering gradients (ε) verses HSA concentration. 90° Rayleigh scattering was performed by titrating *C.albicans* (MRL 3153 grown in RPMI 1640) into filtered 1mM KCl at pH 7.5 supplemented with 0μM, 7.52μM, 53.4μM, 172μM, or 374μM HSA. The increase in emissions intensity at 600nm was recorded after excitation at 600nm. For each HSA concentration, the scattering gradients were determined. The gradients were manipulated by normalizing to zero and multiplying by -1, then these values plotted against HSA concentration and a Hill equation fitted. **Km = 1137.6 ± 6396, n = 1.597 ± 1.2 and the max. was set at 40.** The inset shows the plot of the original data.

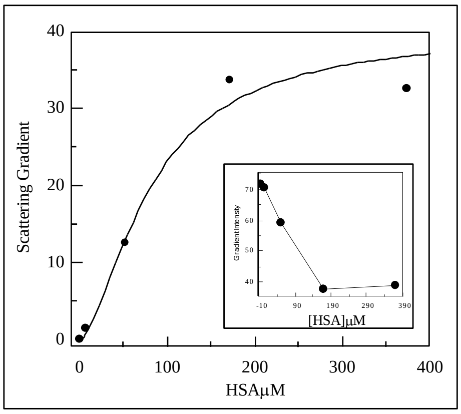

Plot of the scattering gradients (ε) verses HSA concentration. 90° Rayleigh scattering was performed by titrating *C.albicans* (MRL 3153 grown in RPMI 1640) into filtered 1mM KCl at pH 7.5 supplemented with 0μM, 7.52μM, 53.4μM, 172μM, or 374μM HSA. The increase in emissions intensity at 600nm was recorded after excitation at 600nm. For each HSA concentration, the scattering gradients were determined. The gradients were manipulated by normalizing to zero and multiplying by -1, then these values plotted against HSA concentration and a Hill equation fitted. **Km = 1137.6 ± 6396, n = 1.597 ± 1.2 and the max. was set at 40.** The inset shows the plot of the original data.

The effect of calcium ions on the fluorescence of HEXCO labelled *C. albicans*.

The cells were incubated with 100µM HEXCO for 1 hour, with occasional agitiation. After this time, the cells were washed with 1mM KCl + 1mM K^+Hepes, pH 7.5, to remove any unbound HEXCO from the cell suspension. Each experiment was performed in 1mM KCl + 1mM K^+Hepes, pH 7.5. The fluorescence increase upon the addition of 3x10^6 cells/ml was recorded, followed by the effect of 10mM Ca^{2+} on this fluorescence. All measurements were recoreded at 454nm after excitation at 395nm.

Ref., pg3, [B3],

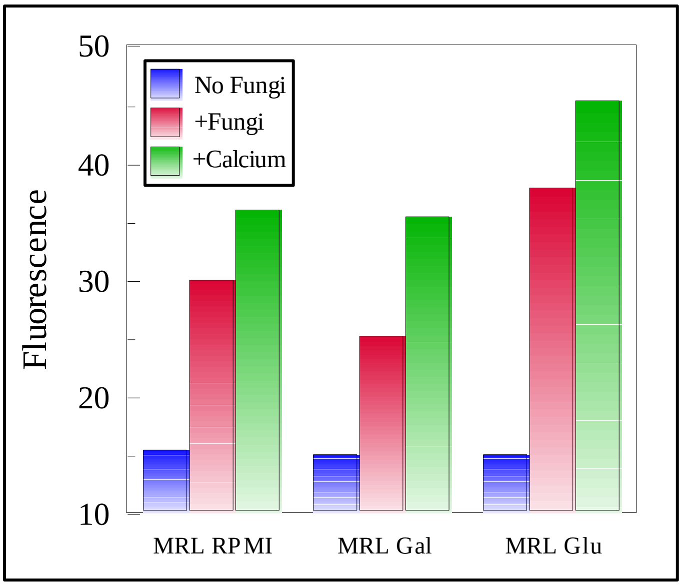

Eppendorf verses Flask as culture receptacle.

The method of *C. albicans* subincubation was examined to establish the that the methods currently employed, namely by means of eppendorf tubes, were valid as well as being convienient and quick.

12 x 1.5ml eppendorf tubes and a 500ml sterile flask were used to incubate GRI 681 in YNB + 500mM glucose, for 18 hours at 37°C.
Each eppendorf tube was filled with 1ml of growth media then inoculated with 1 loop of *C.albicans*. The flask was filled with 250ml of growth media and inoculated with the fungi. Both methods were placed in the incubator for 18 hours at 37°C.

Each experimental investigation was performed using 1mM KCl + 1mM K^+Hepes, pH 7.5.

Assessment Method		Flask	Eppendorf
9-AA Titration	Capacity	168.9 ± 10.5	170.6 ± 6.9
	Kd (cell no. x10^6)	40.5 ± 4.8	36.7 ± 3.9
90° Light Scattering	εs	79.3 ± 2.5	81.0 ± 3.1
ANS Emission Spectra	$\lambda_{max.}$ (Stand. dev. 's')	441.1 nm (0.62)	441.8 nm
	Intensity	218.4	201.6

The effect of 5mM calcium on the fluorescence of cells suspended in 9-AA.

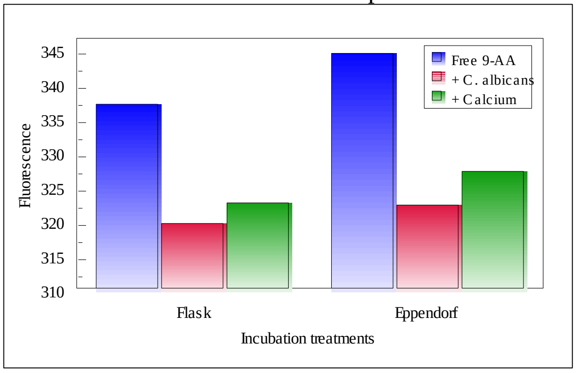

According to the mode of action of changes of the surface electrostatic potential sensed by the probes HEXCO and 9AA – changes of the fluorescence yield take place. In the case of 9AA the fluorescence decreases with addition of the Fungi as the probe binds to the fungal cell surface and with the Ca2+ binding an increase of fluorescence takes place.

Table . The scattering gradients (εs) for each fungal strain in each growth media. Each scattering titration was performed in 1mM KCl + 1mM K^+ Hepes, pH 7.5.

	RPMI 1640	YNB + 500mM Galactose	YNB + 50mM Glucose
MRL 3153	55.4 ± 1.8	51.3 ± 0.9	61.6 ± 1.1
NCPF 3153	70.0 ± 5.4	30.2 ± 1.2	42.3 ± 2.4

Table of scattering gradients (εs) for all the fungi in 1mM KCl, 100mM KCl and 100mM KSCN, ± ≈ 60µM HSA. Ref. pg 95,B2.

**Fungal Strain**	**Growth Medium**	**1mM KCl**		**100mM KCl**		**100mM KSCN**	
		**- HSA [60µM]**	**+ HSA [60µM]**	**- HSA [60µM]**	**+ HSA [60µM]**	**- HSA [60µM]**	**+ HSA [60µM]**
**MRL 3153**	**RPMI 1640**	41.7±3.4	36.8±2.7	36.1±2.5	31.8±1.7	33.4±4.8	17.2±0.7
**MRL 3153**	**YNB + 50mM Glucose**	34.2±2.5	29.5±2.5	32.5±2.8	27.2±2.6	30.3±2.6	25.6±2.4
**MRL 3153**	**YNB + 500mM Galactose**	35.4±1.8	31.5±2.0	37.7±4.1	33.7±2.0	36.2±2.8	32.7±3.7
**NCPF 3153**	**RPMI 1640**	70.0±5.4	56.6±4.9	66.6±4.0	32.0±6.1	60.4±12.9	24.0±1.7
**NCPF 3153**	**YNB + 50mM Glucose**	42.3±2.4	36.0±2.2	40.1±3.6	35.7±3.8	38.3±3.8	33.0±2.6
**NCPF 3153**	**YNB + 500mM Galactose**	30.2±1.2	26.5±1.7	28.2±1.6	24.9±1.3	28.5±2.3	24.1±2.2

Table showing scattering gradients (ε) for all the fungi in 1mM KCl, ± ≈ 60µM HSA. Ref. pg 95,B2, also the % gradient difference.

**Fungal Strain**	**Growth Medium**	**1mM KCl control**	**1mM KCl + 60µM HSA**	**% grad. difference**
**MRL 3153**	**RPMI 1640**	41.7±3.4	36.8±2.7	-11.8 ± 0.9
**MRL 3153**	**YNB + 50mM Glucose**	34.2±2.5	29.5±2.5	-13.7 ± 1.2
**MRL 3153**	**YNB + 500mM Galactose**	35.4±1.8	31.5±2.0	-11.0 ± 0.7
**NCPF 3153**	**RPMI 1640**	70.0±5.4	56.6±4.9	-19.1 ± 1.7
**NCPF 3153**	**YNB + 50mM Glucose**	42.3±2.4	36.0±2.2	-14.9 ± 0.9
**NCPF 3153**	**YNB + 500mM Galactose**	30.2±1.2	26.5±1.7	-12.3 ± 0.8

Table E. The effect of 99mM KCl + 1mM K^+ Hepes on the scattering gradients (εs) for each fungal strain in each growth media, in the presence and absence of HSA.

**Fungal Strain**	**Growth Medium**	**99mM KCl, Control**	**50µM HSA**	**% gradient change**
**MRL 3153**	**RPMI 1640**	47.9 ± 1.9	41.8 ± 2.2	-12.7 ± 0.7
**MRL 3153**	**YNB + 50mM Glucose**	57.4 ± 2.9	48.0 ± 4.3	-16.3 ± 1.5
**MRL 3153**	**YNB + 500mM Galactose**	46.3 ± 1.3	41.4 ± 2.3	-10.6 ± 0.6

The control is 99mM KCl + 1mM K^+ Hepes, (filtered) pH 7.5. Each HSA solution was also prepared with filtered 99mM KCl + 1mM K^+ Hepes, and the pH adjusted to 7.5.

Table F. The effect of 99mM KSCN + 1mM K^+ Hepes on the scattering gradients (εs) for each fungal strain in each growth media, in the presence and absence of HSA.

**Fungal Strain**	**Growth Medium**	**99mM KSCN, Control**	**50µM HSA**	**% gradient change**

**MRL 3153**	**RPMI 1640**	40.3 ± 4.8	20.8 ± 0.9	-48.4 ± 2.0
**MRL 3153**	**YNB + 50mM Glucose**	54.8 ± 2.6	46.3 ± 4.1	-15.5 ± 1.4
**MRL 3153**	**YNB + 500mM Galactose**	48.5 ± 2.8	42.9 ± 4.7	-11.6 ± 1.3

The control is 99mM KSCN + 1mM K^+ Hepes, (filtered) pH 7.5. Each HSA solution was also prepared with filtered 99mM KSCN + 1mM K^+ Hepes, and the pH adjusted to 7.5.

**Fungal Strain**	**Growth Medium**	**1mM KCl control**	**1mM KCl + 60μM HSA**	**% gradient difference**
**MRL 3153**	**RPMI 1640**	41.7±3.4	36.8±2.7	-11.8 ± 0.9
**MRL 3153**	**YNB + 50mM Glucose**	34.2±2.5	29.5±2.5	-13.7 ± 1.2
**MRL 3153**	**YNB + 500mM Galactose**	35.4±1.8	31.5±2.0	-11.0 ± 0.7
**NCPF 3153**	**RPMI 1640**	70.0±5.4	56.6±4.9	-19.1 ± 1.7
**NCPF 3153**	**YNB + 50mM Glucose**	42.3±2.4	36.0±2.2	-14.9 ± 0.9
**NCPF 3153**	**YNB + 500mM Galactose**	30.2±1.2	26.5±1.7	-12.3 ± 0.8

Table of Scat grads. for salts

**Salt Medium**	**Growth Medium**	**MRL 3153**	**NCPF 3153**	**difference**	**difference wrt MRL**
	RPMI 1640	55.4 ± 1.8	70.0 ± 5.4	+14.6	26.3%
**1mM KCl**	YNB + 50mM Glucose	61.6 ± 1.1	42.3 ± 2.4	-19.3	31.3%
	YNB + 500mM Galactose	51.3 ± 0.9	30.2 ± 1.2	-21.1	41.1%
	RPMI 1640	47.9 ± 1.9	66.6 ± 4.0	+18.7	39.0%
**99mM KCl**	YNB + 50mM Glucose	57.4 ± 2.9	40.1 ± 3.6	-17.3	30.0%
	YNB + 500mM Galactose	46.3 ± 1.3	28.2 ± 1.6	-18.1	39.1%
	RPMI 1640	40.3 ± 4.8	65.2 ± 4.1	+24.9	61.8%
**99mM KSCN**	YNB + 50mM Glucose	54.8 ± 2.6	38.3 ± 3.8	-16.5	30.1%
	YNB + 500mM Galactose	48.5 ± 2.8	28.5 ± 2.3	-20.0	41.2%

Effect of Culture conditions on 0.25μM 9-AA fluorescence of GRI 681 grown in glucose.

Culture conditions: 4 x 500ml bottles were prepared with 250ml of YNB + 50mM glucose, each was inoculated using 10 loops each of *C. albicans*; GRI 681. The prepared bottles were incubated at 37°C, for 186hrs from which samples were extracted and investigated after 18hrs and 186hrs.

1. Control, no aggitation - 'anaerobic'.
2. + 2mg/ml HCO_3^-, no aggitation - 'anaerobic'.
3. + filtered, CO_2 stripped air bubbled through, 'aerobic'.

4. + filtered, ordinary air bubbled through, + 2mg/ml HCO_3^-, 'aerobic'.

After the appropriate incubation, the extracted cells were washed in low salt (1mM KCl + 1mM K^+Hepes) medium pH 7.5 and used to perform 9-AA cell titrations. The effect of the cellular additions on the fluorescence of 0.25μM 9-AA, were measured at 455nm after excitation at 400nm. For each titration, low salt medium at pH 7.5, was used. For the 18 hr incubation, up to 213 x 10^6 cells were used and for the 186 hr incubation, up to 185 x 10^6 cells were used.

Culture Conditions	18 Hour Incubation		186 Hour Incubation	
	Capacity	Kd (cell no.x 10^6)	Capacity	Kd (cell no.x 10^6)
1	170.6 ± 6.9	36.7 ± 3.9	407.7 ± 39.7	39.3 ± 8.1
2	245.7 ± 28.7	35.7 ± 5.4	-	-
3	163.9 ± 8.5	48.0 ± 5.3	405.9 ± 33.8	38.1 ± 6.9
4	232.7 ± 6.7	63.3 ± 3.6	373.4 ± 13.7	37.9 ± 3.1

Results for bottle 2 are unreliable - only 6 x 10^6 cells were available and for 186 hr inc., no cells available.

The effect of 5mM calcium on the 9-AA fluorescence of *C. albicans*, after 18 hours of the four treatments.

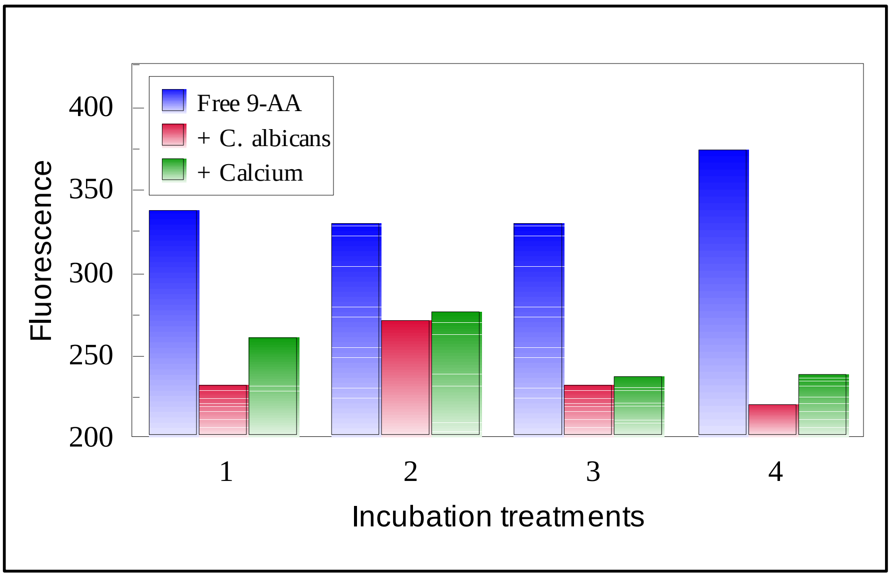

The effect of 5mM calcium on the 9-AA fluorescence of *C. albicans*, after 186 hours of the four treatments.

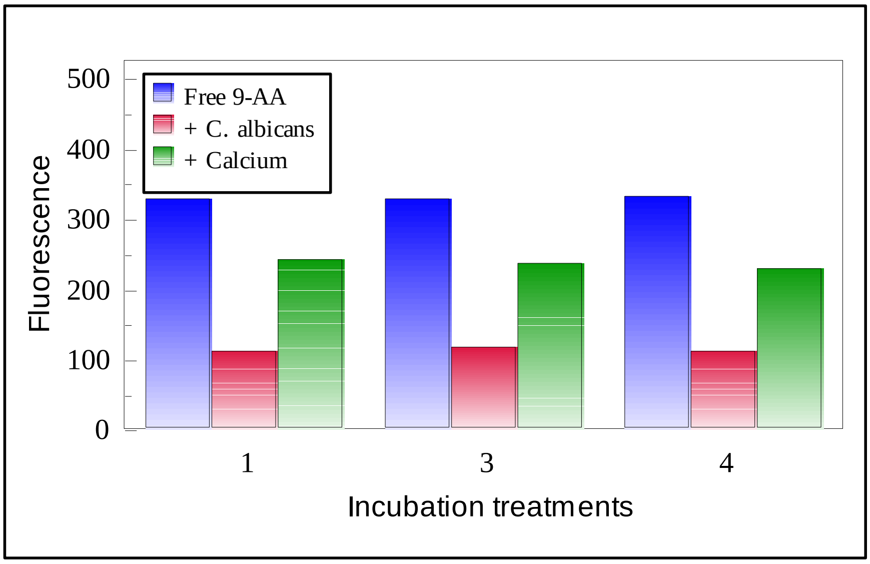

Scattering data.

After the appropriate incubation, the extracted cells were washed in low salt (1mM KCl + 1mM K^+Hepes) medium, pH 7.5. The effect of cellular additions on 90° Rayleigh-Debye light scattering, was assessed at 600nm. For each titration, filtered, low salt medium at pH 7.5, was used. For the 18 hr incubation, up to 17 x 10^6 cells were used and for the 186 hr incubation, up to 12 x 10^6 cells were used.

Culture Conditions	18 Hour Incubation (εs)	186 Hour Incubation(εs)
1	81.0 ± 2.1	117.9 ± 0.7
2	174.7 ± 3.7	-
3	67.8 ± 0.4	110.4 ± 1.1
4	56.2 ± 1.1	73.8 ± 0.7

HSA Candida Interactions

Figure showing both fits, low and high, to all the data points for the HSA titration into HEXCO labelled MRL 3153 (grown in RPMI 1640) suspended in 1mM KCl.

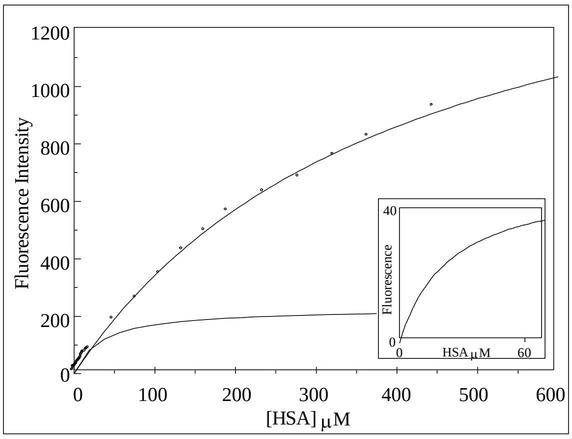

The effect of increasing HSA concentration on HEXCO labelled C. albicans (MRL 3153 grown in RPMI 1640). Cells were supplemented with 100μM HEXCO and incubated for 1 hour at 37°C with occasional agitation. After removal of unbound HEXCO, aliquots of HSA were titrated into 7.5x10^6 labelled cells/ml suspended in 1mM KCl at pH 7.6. The increase in fluorescence emission was

recorded at 454nm after excitation at 395nm. The data fits shown demonstrate two binding curves, one with Kd = 17.1 ± 1.9 and Capacity = 98.7 ± 7.6 and the other with Kd = ± and Capacity = ±. these are believed, respectively, to indicate specific receptor binding and none specific (hydrophobic) binding. The inset explains clearly the fit to the low Kd and Capacity values.

Note: pg 4, [B3]; says that at low [HSA], fungi take a little time to react but at high [HSA] it is instantaneous. FN reacts like lo [HSA]; 'relatively' slowly.

--

Figure showing the effect of HSA on the scattering of MRL 3153 grown in RPMI 1640, in a medium containing 1mM KCl.

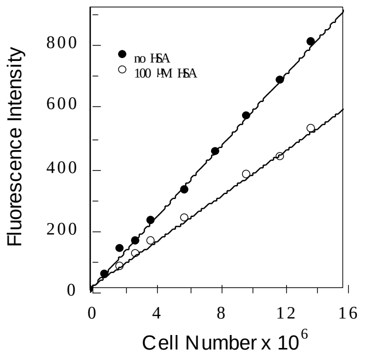

--

Older Pics.

Figure showing both fits, low and high, to all the data points for the HSA titration into HEXCO labelled MRL 3153 (grown in RPMI 1640) suspended in 1mM KCl. (Picture above with frame and underneath without)

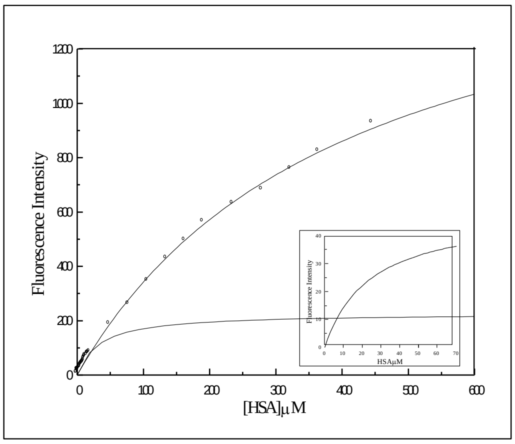

Figure showing both fits, low and high, to all the data points for the HSA titration into HEXCO labelled MRL 3153 (grown in RPMI 1640) suspended in 1mM Kcl.

Effect of titrating high [HSA] into HEXCO labelled MRL 3153 grown in RPMI 1640, suspended in 1mM KCl.

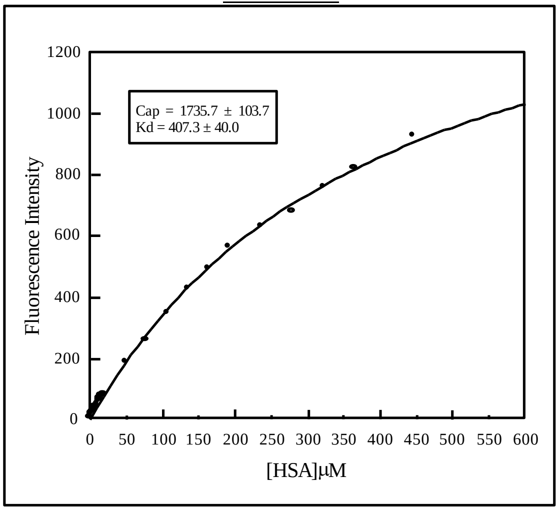

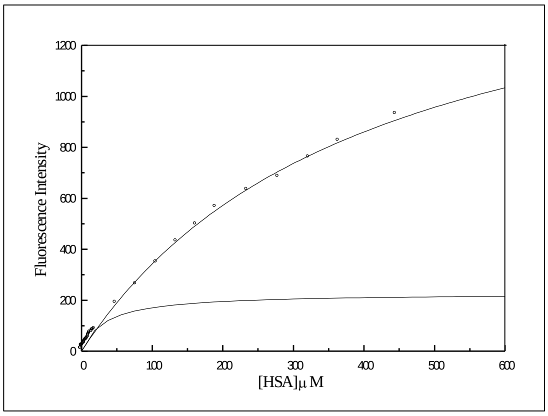

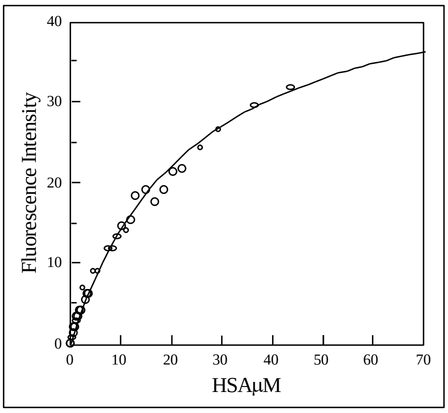

fit for low Kd & Cap Kd =17.1 ± 1.9 & Cap = 98.5 ± 7.6

Figure showing the effect of low HSA concentration on the fluorescence intensity of HEXCO labelled MRL 3153 grown in RPMI 1640. The cells were labelled with 100μM HEXCO for 1 hour at 37°C with occasional agitation. $3x10^6$ cells/ml were suspended in a medium containing 1mM KCl at a pH of 7.6.

Aliquots of HSA were titrated into the cell suspension with gentle agitation, then the change in fluorescence intensity recorded at 454nm after excitation at 395nm.

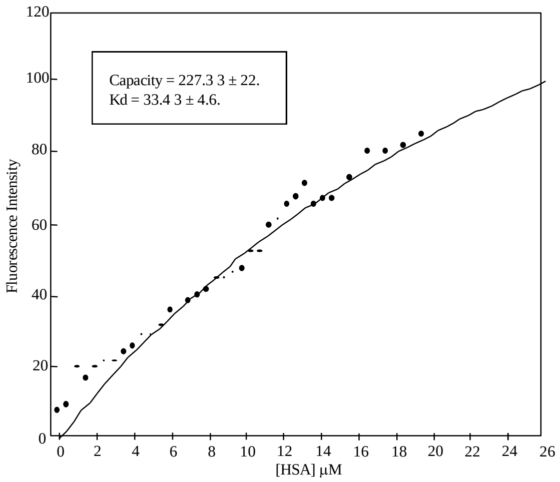

The effect of low HSA concentration on HEXCO labelled MRL 3153 RPMI 1640 at pH 7.6.

The effect of [HSA] on MRL RPMI- the edited, first 36 data points.

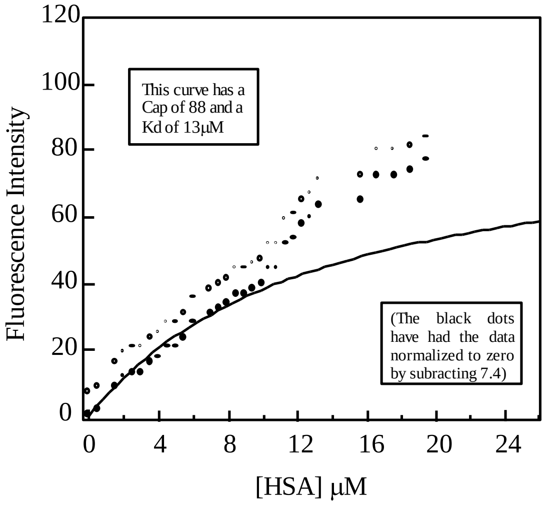

Table showing a summary of the trends obtained from the single ligand binding equations.

Strain and growth medium	± DTT reduction	0 – 20 μM HSA		0 – 446 μM HSA	
		Capacity	Kd μM	Capacity	Kd μM
MRL 3153 in rpmi*	-	98.5 ± 7.6	17.1 ± 1.9	1547.0 ± 93.9	364.1 ± 36.2
GDH 2346 in rpmi	-	- 10.2% ± 1.02	+ 35.2% ± 6.3	- 22.1% ± 2.1	- 40.5% ± 7.7
GRI 681 in rpmi	-	+ 153.4% ± 33.2	+ 325.9% ± 87.9	+ 8.0% ± 1.0	+ 15.7% ± 3.2
NCPF 3153 in rpmi	-	+ 43.3% ± 5.23	+ 125.9% ± 23.7	+ 24.1% ± 2.1	+ 41.4% ± 5.7
MRL 3153 in galactose†	-	- 39.1% ± 5.3	+ 19.0% ± 3.3	-	-

* the average of six experiments.

† 0 – 90 μM HSA

The results shown are relative to those values for MRL 3153 grown in RPMI 1640. Data (both for Capacity and Kd) was manipulated by subtracting the MRL rpmi value to obtain the difference, then dividing the difference by the MRL rpmi value and multiplied by 100 to get the final %. The ± value was obtained by dividing the original ± value by the original value and multiplied by 100 to get a %, then finding this % of the final %.

e.g.: for Cap = a ± b, Kd = c ± d,

$$\text{a or c - MRL rpmi} = \text{e}, \left[\frac{\text{e}}{\text{MRL rpmi}}\right] \times 100 = \textbf{final \%}.$$

$$\left[\frac{\text{b or d}}{\text{a or c}}\right] \times 100 = \text{f}, \left[\frac{\text{f}}{100}\right] \times \text{final \%} = \textbf{final} \pm.$$

For low HSA concentration ranges in RPMI 1640; Capacity: GRI 681>NCPF 3153>MRL 3153>GDH 2346
Kd: GRI 681>NCPF 3153>GDH 2346>MRL 3153
For High HSA concentration ranges in RPMI 1640; Capacity: NCPF 3153>GRI 681> MRL 3153>GDH 2346
Kd: NCPF 3153>GRI 681> MRL 3153>GDH 2346

Generally fungi with high capacity values have a high value for Kd and fungi with low capacity values have low values for Kd. This indicates that different strains in the same growth medium bind HSA differently. This implies that at low HSA concentrations a more specific mechanism, which depends on strain, is happening, whereas at high HSA concentrations, a less discriminate process occurs. So at low HSA concentration ranges, GRI 681 has the most individual areas of receptor mediated binding but each of these areas are weakly populated, followed by NCPF 3153 as the next most homogeneous strain. MRL 3153 and GDH 2346 are more heterogeneous. At high HSA concentration ranges, again NCPF 3153 and GRI 681 show the greatest homogeneity and MRL 3153 and GDH 2346 are more heterogeneous, although the relative differences are not as profound as with low HSA concentrations.
